# Ambivalent Roles of Oxidative Stress in Triangular Relationships among Arthropod Vectors, Pathogens and Hosts

**DOI:** 10.3390/antiox11071254

**Published:** 2022-06-25

**Authors:** Emmanuel Pacia Hernandez, Anisuzzaman, Md Abdul Alim, Hayato Kawada, Kofi Dadzie Kwofie, Danielle Ladzekpo, Yuki Koike, Takahiro Inoue, Sana Sasaki, Fusako Mikami, Makoto Matsubayashi, Tetsuya Tanaka, Naotoshi Tsuji, Takeshi Hatta

**Affiliations:** 1 Department of Parasitology and Tropical Medicine, Kitasato University School of Medicine, 1-15-1 Kitasato, Minami, Sagamihara 252-0374, Kanagawa, Japan; ephernandez4@alum.up.edu.ph (E.P.H.); hkawada@med.kitasato-u.ac.jp (H.K.); kwofiek@gmail.com (K.D.K.); dladzekpo.vip@tmd.ac.jp (D.L.); mikami@kitasato-u.ac.jp (F.M.); tsujin@med.kitasato-u.ac.jp (N.T.); 2 Department of Parasitology, Faculty of Veterinary Science, Bangladesh Agricultural University, Mymensingh 2202, Bangladesh; zaman.a.bau@gmail.com (A.); aalimpara@bau.edu.bd (M.A.A.); 3 Department of Molecular and Cellular Parasitology, Kitasato University Graduate School of Medical Science, 1-15-1 Kitasato, Minami, Sagamihara 252-0374, Kanagawa, Japan; koike.yuki@st.kitasato-u.ac.jp (Y.K.); dm21005@st.kitasato-u.ac.jp (T.I.); mm21025@st.kitasato-u.ac.jp (S.S.); 4 Department of Parasitology, Noguchi Memorial Institute for Medical Research, College of Health Sciences, University of Ghana, Legon, Accra P.O. Box LG 581, Ghana; 5 Department of Environmental Parasitology, Tokyo Medical and Dental University, 1-5-45 Yushima, Bunkyo-Ku, Tokyo 113-8510, Japan; 6 Department of Veterinary Immunology, Graduate School of Veterinary Sciences, Osaka Metropolitan University, Izumisano 598-8531, Osaka, Japan; matsubayashi@omu.ac.jp; 7 Laboratory of Infectious Diseases, Joint Faculty of Veterinary Medicine, Kagoshima University, 1-21-24 Korimoto, Kagoshima 890-0065, Japan; k6199431@kadai.jp

**Keywords:** ticks, mosquitoes, oxidative stress, ROS, microbiome, acaricide, insecticide resistance

## Abstract

Blood-feeding arthropods, particularly ticks and mosquitoes are considered the most important vectors of arthropod-borne diseases affecting humans and animals. While feeding on blood meals, arthropods are exposed to high levels of reactive oxygen species (ROS) since heme and other blood components can induce oxidative stress. Different ROS have important roles in interactions among the pathogens, vectors, and hosts. ROS influence various metabolic processes of the arthropods and some have detrimental effects. In this review, we investigate the various roles of ROS in these arthropods, including their innate immunity and the homeostasis of their microbiomes, that is, how ROS are utilized to maintain the balance between the natural microbiota and potential pathogens. We elucidate the mechanism of how ROS are utilized to fight off invading pathogens and how the arthropod-borne pathogens use the arthropods’ antioxidant mechanism to defend against these ROS attacks and their possible impact on their vector potentials or their ability to acquire and transmit pathogens. In addition, we describe the possible roles of ROS in chemical insecticide/acaricide activity and/or in the development of resistance. Overall, this underscores the importance of the antioxidant system as a potential target for the control of arthropod and arthropod-borne pathogens.

## 1. Introduction

Every year more than 700,000 individuals die from diseases transmitted by hematophagous arthropods, of which malaria, dengue, human African trypanosomiasis, leishmaniasis, Chagas disease, yellow fever, Japanese encephalitis, onchocerciasis, and tick-borne encephalitis are particularly detrimental [1]. Hematophagous arthropods such as mosquitoes, triatomine bed bugs, sandflies, blackflies, midges, and ticks derive their nutritional requirement from blood feeding. While some of them are facultative blood feeders such as the female mosquitoes that need blood to trigger oogenesis; others, such as ticks, are obligate blood feeders that solely depend on blood meals from their hosts for their propagations, molting, development, and survival [2,3,4,5,6,7]. Most of them ingest huge amounts of blood in a single meal ranging from three to ten times their body weight to up to several hundred times their unfed body weight [8,9]. Due to this unique feeding behavior, these arthropods have become efficient bridging agents between vector-borne pathogens and their hosts. Therefore, the control of these vectors could also lead to the control of these diseases. Among blood-feeding arthropods, mosquitoes and ticks are recognized as the most important vectors of pathogens affecting animals or humans. Of the arthropod-borne diseases, only the mosquito-borne pathogens cause high morbidity and mortality globally and the collective global burden of mosquito-borne diseases is not less than that of AIDS. Malaria, a mosquito-borne protozoan disease, alone attributes to 45 million disability-adjusted life years (DALYs) per year and is considered one of the “big three” along with AIDS and tuberculosis [10]. On the other hand, ticks are only second to mosquitoes in terms of their potential to transmit a wide array of devastating pathogens, posing a global threat to both human and animal health. Therefore, the control of these vectors is of public health and veterinary concern [11].

Blood is an excellent source of nutrients for hematophagous arthropods; however, its components including heme, iron, amino acids, and even water could be deleterious for the attacking vectors themselves. Heme and free iron lead to the formation of reactive oxygen species (ROS), resulting in oxidative stress. Previous works of literature have already shed some light into the mechanisms of how vectors are engaged in the neutralization of oxidative stresses for their survival [8,12,13,14]. In this review, we describe the roles of ROS in mosquitoes and ticks, particularly in vector competency and the development of insecticide/acaricide resistance.

## 2. ROS Generation and Oxidative Stress in Arthropods

ROS are free radicals that are highly unstable and reactive and contain one or more unpaired electrons. They are naturally produced in all cells and organisms and are crucial in several metabolic processes including cell signaling, cellular proliferation, and transcription regulation [15,16]. A tight balance between ROS production and its neutralization supports healthy life and vector potentials and ameliorates the effects of insecticides/acaricides. However, an imbalance between ROS production and antioxidant activities results in cellular damage, a phenomenon known as oxidative stress, which is highly detrimental to the life of any organism, including hematophagous vectors.

### 2.1. Blood Meal and the Fate of Heme within Arthropods

Blood is an excellent source of nutrients, rich in proteins; it also contains the necessary carbohydrates, salts, and lipids that can supply the needs of the arthropod as well as its embryo development [17,18,19,20]. Hemoglobin and albumin, the most abundant proteins in the blood, make up almost 80% of the total proteins of blood and are greatly utilized by hematophagous arthropods and blood-dwelling parasites [21]. When hemoglobin is broken down during hematophagy, it results in the liberation of large amounts of heme. Heme itself is capable of promoting the production of ROS [14,22]. In vivo study demonstrates that most of the heme ingested is not absorbed but readily excreted in the feces through several mechanisms. In hemipterans, the lipid membranes in the gut epithelium catalyze the formation of crystalline heme aggregates known as hemozoin. In the mosquitoes, the heme is trapped in the peritrophic matrix of the gut. Ticks, on the other hand, digest blood intracellularly [23,24,25,26], wherein the liberated heme is trapped in hemosomes, specialized organelles in tick-gut cells designated to trap the liberated heme [8,22]. Although the gut of hematophagous arthropods efficiently tends to thwart the absorption of heme, a significant amount of heme is retained within and is capable of inducing oxidative damage at the cellular level and causes lipid peroxidation [12,14]. A mechanism to degrade heme and reduce its toxic effects is also present within vectors. Although seemingly beneficial, it can cause another problem by releasing highly reactive free Fe^2+^, which may trigger the Fenton reaction, ultimately resulting in accelerated ROS production, and thus, eventually exacerbating oxidative stress [12]. However, this phenomenon is not observed in ticks since they lack the heme oxygenase gene [27]. In sandflies and *Aedes aegypti*, hemoglobin digestion intermediates also induce oxidative stress upon contact with the gut epithelium [16,28]. Iron is also acquired from the serum from the hosts’ ferritin, transferrin, and other iron-binding proteins found in the host blood [13].

Hard ticks are pool feeders, and they attach for a long period that can range for 3–12 days depending on the species and developmental stage of life cycle. Interestingly, the attachment period may extend up to 60 days when they attach to a reptile host [3,5,6,29,30,31]. During feeding, ROS are also generated from the hosts’ eosinophils, neutrophils, and macrophages through the lesion brought about by blood feeding [5,30,32,33]. In blood-feeding arthropods such as mosquitoes and ticks, the control of oxidative stress from heme and iron toxicity, which includes postprandial downregulation of ROS production in insect cells, matrix peritrophins, heme catabolism, and iron chaperones and shuttling, has already been thoroughly discussed in previous works [12,17,22].

### 2.2. Other Biological Sources of ROS

A variety of cellular developmental and metabolic activities and other biological events can lead to the generation of ROS (Table 1). Increased malondialdehyde (MDA) concentrations, an indicator of oxidative stress, were also observed during embryonic development as well as larval maturation of the ticks such as *Rhipicephalus microplus* and *Haemaphysalis longicornis* [34,35]. In mosquitoes, an increase in metabolic activities, including oogenesis, also results in the generation of ROS and these ROS accumulate over time and can affect the fecundity of mosquitoes [36]. Since mosquitoes are flying insects, ROS generation is further accelerated by the increased mitochondrial activity of the flight muscles [37]. Although it is not proven in blood-feeding arthropods, studies in insect models such as *Drosophila* indicate that a high production of ROS in the insect nervous system results in the ROS-mediated decline of neuron survival [16].

## 3. Vector Competency and Oxidative Stress

Vector competence (also termed vector potential) refers to the ability of arthropods to transmit pathogens, which is greatly influenced by the genetic and/or other intrinsic factors of arthropod vectors [46]. Additionally, it is also governed by the factors exerted by hosts themselves during pathogen inoculation, development, and propagation in particular hosts. During an infection, ROS have pivotal roles in the triangular relationship among vectors, pathogens, and hosts and may influence the triad either positively or negatively. A pluripotent molecule isolated from the salivary glands of *H. longicornis,* called longistatin [47], plays a central role in the feeding and development of ticks [3,4,9,48] and has been elegantly shown to ameliorate cellular ROS production in human endothelial cells [5], making it a key molecule in the survival of hard ticks. On the other hand, the acquisition of pathogens into a vector also induces modification of the normal ROS production resulting in oxidative stress to arthropod cells, which ultimately is being utilized by hematophagous arthropods to eliminate invading pathogens. Therefore, vector competence largely depends on a smart balance of the ROS that ensures the entrance, survival, and proliferation of pathogens into a vector. At the same time, maintenance of an optimum level of ROS is essential for the assurance of survival of the arthropods themselves to allow the feeding behavior that eventually ensures pathogen transmission [32,49,50,51].

### 3.1. ROS and Arthropod’s Innate Immunity

**Figure 1:**
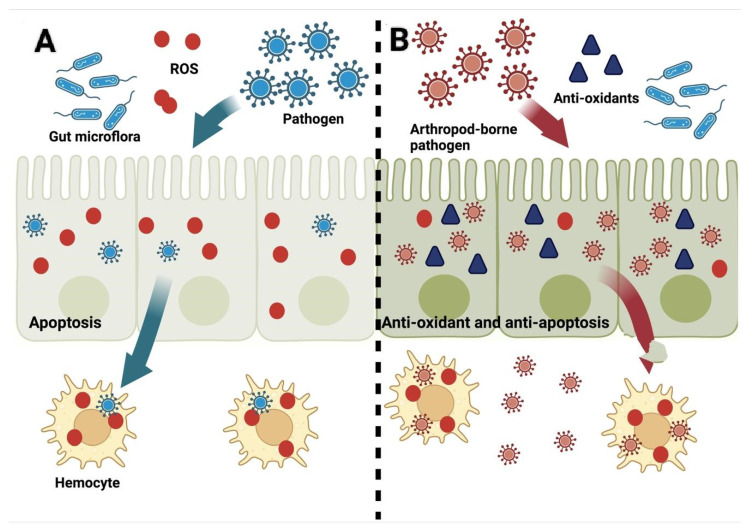
Schematic diagram of the life cycle of the pathogen (**A**) after infection of the arthropod versus the arthropod-borne pathogen’s (**B**) life cycle and its interaction with ROS. Created with Biorender.com (accessed on 10 May 2022).

To ensure their own survival and existence, arthropods use ROS to eliminate invading pathogens as well as to mount a better immune response during infection (Figure 1A) [52,53,54]. *Anopheles gambiae,* for example, can survive better at higher levels of systemic ROS when challenged with *Micrococcus* and *Escherichia*. Furthermore, the supplementation of antioxidants in the diet results in significantly higher mortality during bacterial infection [55], indicating that ROS and oxidative stress play a critical role in the arthropods’ survival during the acquisition and transmission of infections. On the other hand, a *Plasmodium* refractory strain of *Ano. gambiae* was observed to be in a chronic state of oxidative stress, while the same parasite would survive if antioxidants were provided in the diet [8,40]. The same effects of oxidative stress are observed in the *Rh.* *microplus* (BME26) cell line. During infection with *Rickettsia ricketsii* or exposure to heat-killed microorganisms, upregulation of genes encoding for ROS production was observed, while antioxidant genes were downregulated [56]. Oxidative burst by macrophages efficiently eliminates pathogens basically by ROS, which are either toxic to the pathogen or work together with hydrolases, reactive nitrogen species, and the NADPH oxidase system (NOX). Supporting the above notion, bacterial infections have been shown to increase ROS in the ticks’ hemocytes [39]. ROS play a role to block pathogen transmission by melanotic encapsulation, where invading pathogens are encapsulated to help the prevention of transmission. Melanotic encapsulation of *Plasmodium* has been shown in *Ano. gambiae.* The melanocytic capsule of the refractory strains of *Ano. gambiae* constructed around *Plasmodium* can block parasite development in the mosquitoes’ midgut and the strain was observed to have higher levels of ROS [40]. On the other hand, in mosquitoes, ROS also act as a signaling molecule for the mitogen-activated protein kinase (MAPK)–dependent cascade and phosphatidylinositol 3-kinase (PI3K)/Akt-dependent pathway, which has been shown to regulate innate immunity and affects the physiology and development of the malarial parasite [41,42].

### 3.2. ROS after the Establishment of Infection in Hosts

While ROS are an important component of defense by the host against the pathogen, ROS are also generated during the establishment of a pathogen within a host and continue to be produced throughout the progression of the disease, for example, flaviviruses are known to induce the production of ROS, which are linked to apoptosis and are, thus, involved in the killing of infected cells together with the pathogen and eventually support the survival of the remaining non-infected cells. Through the production of ROS, infected individuals battle against pathogens to eliminate invading microbes in the early stage of invasion to prevent the progression of damage to the adjacent cells caused by the pathogens themselves and by the triggered inflammatory insults as well (Figure 1A) [57,58]. Therefore, this cellular response could affect the vectorial capacity of the arthropods. However, the coevolution of the arthropods with the pathogens carried by them has led to their coadaptation with each other’s immune responses (Figure 1B). Pathogens have devised various protective shields to evade host responses to ensure their transmission, colonization, and survival in a hostile environment within vertebrate hosts, until either the recovery or the death of the host [49,50,59].

During dengue virus (DENV) infection, apoptosis is the usual outcome [60]. Apoptosis is usually brought about by the production of viral proteins, which disrupts the function of the endoplasmic reticulum (ER), resulting in the accumulation of misfolded and unfolded proteins. The presence of these misfolded and unfolded proteins activates the unfolded protein response (UPR). Even with the UPR, the mitigation of the effect of ER stress may not be addressed within a specific time and will still result in apoptosis [61,62]. However, in a mosquito cell line, it was found that mosquito cells were neither severely damaged nor subjected to apoptosis, rather the infection persisted in this setting, and ROS were detected. Interestingly, a p53 paralogue was upregulated during infection. The p53 is a transcription factor that selectively transcribes the catalase gene, which alleviates ROS accumulation within the cells, therefore reducing the rate of apoptosis (Figure 1B). In experiments that reduced the expression of the *p53* gene, ROS accumulated in the infected cells [60]. In *Ae. aegypti*, knockdown of the catalase gene also resulted in reduced oviposition and lifespan with H_2_O_2_ challenge and reduced virus titer in the midgut upon infection with DENV [63]. Aside from the catalase activity, glutathione S-transferase (GST) activity was also higher in DENV-infected cells. GST suppression resulted in an earlier release of superoxide ions and higher cell mortality. Interestingly, this upregulation was not observed in mammalian cells infected with the same virus, indicating that this phenomenon may be limited to only mosquito cells [64]. Besides GST activity, an additive anti-apoptotic activity was observed due to the upregulation of the inhibitor of apoptosis (IAP) [61]. ROS and oxidative stress are also believed to be controlled by the proper refolding of misfolded proteins, and this is usually achieved through the production of the X-box binding protein 1 (XBP1), which is presumed to be a critical transcription factor for various chaperones, including the *BiP/GRP 78* mRNA [62]. In contrast, West Nile virus, another flavivirus, also induces ROS production. However, the exact mechanism of this ROS production is still unknown. Mosquito cells infected with this virus-induced upregulation of Nrf2- and NRF1-mediated antioxidant genes, eventually result in elevated reduced glutathione (GSH) levels. This ultimately increased the oxidative capacity of the cells to withstand the oxidative stress elicited by the virus infection [65].

In contrast, transfection with the nonstructural protein 1 of the flavivirus, e.g., tick-borne encephalitis virus, induces oxidative stress in HEK293T cells and activates the antioxidant defense of these cells [66]. Moreover, in tick cells, the knockdown of the antioxidant *GST* molecule with subsequent infection of the Langat virus, another member of Flaviviridae, resulted in increased mortality, decreased proliferation, and decreased viral titer [67]. Furthermore, infection of LGTV in tick cells indicates a possible correction of the protein folding as seen by the upregulation of chaperone proteins, specifically heat shock proteins (HSPs) 90 and 70 [68,69]. These HSPs help in the refolding of misfolded proteins or are related to the degradation of terminally misfolded proteins to prevent protein aggregation, thereby creating an anti-apoptotic environment within cellular niches [58,70]. This corrective response was also observed in *Anaplasma phagocytophilum* infection in ticks and tick cells, wherein HSP20, HSP70, and HSP90 expressions have been upregulated [58,71,72]. Metabolomics also indicates that terminally misfolded proteins tend to prevent ER stress and apoptosis. Accordingly, *HSP70* knockdown decreases *Ana. phagocytophilum* titer in ticks [71].

Furthermore, *Ana. marginale* infection upregulated genes closely related to the generation of antioxidants. Simultaneous knockdown of catalase, glutathione peroxidase, and thioredoxin together with oxidative resistance 1 gene favored the colonization of *Ana. marginale* in BME26 cells, strongly supporting that the oxidant response is involved in the control of infection and the maintenance of cell survival [56]. Additionally, mitochondrial ROS production also increases in response to *Ana. phagocytophilum* to control the parasite. Conversely, to maintain the parasite’s fitness and maintain the infection, other alternative ROS production and apoptosis pathways are also inhibited [73].

Another group of molecules that are also upregulated during infection are selenoproteins. They are a group of proteins that both catalyze and regulate several redox reactions [38]. It has been proposed that pathogens can induce the production of selenoproteins that not only allow their proliferation and transmission but also play a key role in pathogen acquisition. In *Borrelia burgdorferi* infection, Salp25D, a tick selenoprotein, is utilized against the oxidative stress from the inflammatory process at the biting site [32]. Knockdown of selenoprotein M reduces the titer of *Ana. marginale* and inhibits the development of the infective stage of *Ana. marginale* [74]. Selenoprotein P (SelP) is upregulated in the salivary glands of the *Ri. parkeri*–infected ticks to ameliorate oxidative stress during feeding. Furthermore, the knockdown of *SelP* genes also reduced the transovarial transmission of pathogens [52]. In addition to the direct control of ROS through antioxidant enzymes, the generation of ROS is also controlled by regulating free cations such as iron, which augments ROS productions. Ferritins that sequester free iron are upregulated in *Dermacentor variabilis* during *Escherichia coli* infection [13]. Bacterial iron-binding proteins could also sequester iron in the blood meal, which is expressed in the infective stage of *Ana. phagocytophilum* [75].

### 3.3. ROS and Arthropod Microbiome

In this review, microbiome refers to the overall genome of microorganisms in a certain niche, which has been shown to shape the phenome [76,77]. Therefore, attaining a balance between the natural microbiota and potential pathogens is very crucial. One way to maintain this balance is through the dual oxidase (Duox)–dependent ROS generation system [16]. Duox is a protein that mainly functions in the generation of ROS; however, the Duox-ROS pathway remains inactive unless proliferation occurs and bacteria come in contact with the mucosal barrier. In this manner, ROS produced from the Duox pathways attack invading pathogens through the mucosal barrier, particularly by H_2_O_2._ These attacks can disrupt the tyrosine phosphorylation network of invading pathogens and, thereby, reduce their fitness. Enterobacteria dominate in the midgut by maintaining gut homeostasis [78,79,80], and these bacterial species are adapted to survive within blood-feeding arthropods (e.g., ticks) as they are resistant to ROS killing. Since enterobacterial species are ROS-generating bacteria, during blood feeding, the gut environment would favor the growth of these bacterial species, overcoming the possible proliferation of *Plasmodium* and other pathogenic organisms [43,81,82,83]. Challenge with pathogens, therefore, accelerates the expansion of bacterial populations during blood feeding and they escape the constraints of the Duox system. One way for the natural microbiota to escape the Duox system is by avoiding contact with the gut epithelium. To achieve this, bacteria are encaged in a blood bolus during blood feeding. The formation of a dityrosine network (DTN) on the luminal surface of the gut epithelium also makes it difficult for the soluble immune mediators to penetrate the blood bolus; thus, the microbiota avoids activation of the immune responses [84]. The protective effects of this DTN are not only beneficial for the microbiota but also for the *Plasmodium* parasite [85]. The same DTN is also found in ticks and has been proved to maintain *B. burgdorferi* infection.

Furthermore, in ticks, some pathogenic organisms have already adapted to this strategy by altering their transcription mechanism and ameliorating the antioxidant mechanisms, including selenoproteins, to maintain the favorable levels of ROS that allow for their survival and growth. The knockdown of the *SelP* gene increases oxidative stress, thus, decreasing *Ri. parkeri* loads and increasing levels of *Francisella*-like symbionts such as *Candidatus* Midichloria mitochondrii and other bacteria [43,52].

## 4. ROS and Chemical Control of Arthropods

**Figure 2:**
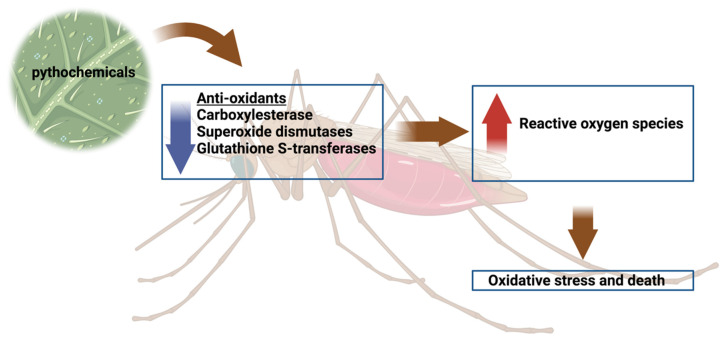
Schematic diagram on the activity of the phytochemicals and its possible mode of activity to cause mortality. Created with Biorender.com (accessed on 10 May 2022).

The metabolism of xenobiotics including acaricides and insecticides also leads to the generation of ROS [16]. Enzymes involved in the detoxification of these acaricides are also known as antioxidants, including glutathione S-transferase, cytochrome P450 monooxygenases, and some esterases. Higher expression or activities of these enzymes are involved with some resistant strains in both mosquitoes and ticks [44,86,87]. Permethrin-resistant strains of *Ano. gambiae* were shown to have higher ROS production rates and the increased ROS production in turn resulted in increased *GST* and *catalase* gene expression [44]. The same observations were reported in DDT-resistant *Ano. arabiensis* and *Ano. funestus,* wherein increased catalase and glutathione peroxidase activity was observed, indicating that resistant strains of mosquitoes need a higher capacity for coping with oxidative stress [88]. During every single blood meal, a transient decrease of oxidative stress was observed in mosquitoes. Increased expression and activity of antioxidant enzymes after multiple bouts of blood feeding is essential to maintain the homeostasis of DDT- and permethrin-resistant phenotypes of mosquitoes [88]. Disruption of the oxidative stress, e.g., elevated oxidative stress or reduced capability of minimizing oxidative stress, increases the susceptibility of DDT-resistant *Ano. gambiae* to DDT [89]. Additionally, some phytochemicals extracted from plants can augment ROS production. When *Culex quinquefasciatus* is exposed to the extracts of the medicinal plant, *Stachytarpheta jamaicensis,* it can lead to the generation of a high amount of ROS, resulting in the death of their larvae [45]. Plant extracts can decrease the levels of antioxidative enzymes in mosquitoes, for example, phytochemicals reduce the levels of carboxylesterases and superoxide dismutases in *Ae. aegypti* larvae (Figure 2) [90,91]. In the ticks, a negative correlation between GST enzyme expression and different phytochemical concentrations was observed, indicating the ability of these phytochemicals to induce oxidative stress in ticks [92].

## 5. Conclusions

Mosquitoes and ticks are considered to be the most important vectors in the transmission of human and animal disease-causing pathogens. The vast array of pathogens includes viruses, bacteria, protozoa, and nematodes. Pathogens’ acquisition and transmission largely depend on the ability of the pathogens to evade not only the arthropod’s immune response but also their physiology. ROS are an integral part of the cellular physiology of any organism. In hematophagous arthropods, high amounts of ROS are produced by heme released during the digestion of blood meals. Moreover, several biological activities such as embryonic and larval development and flight activity augment ROS production. ROS have dual functions and are essential mediators of varieties of biological functions, including immune responses, cell signaling, and maintenance of the natural microbiome. ROS are also crucial in maintaining the natural microbiota in ticks. On the other hand, high amounts of ROS could result in redox imbalance, causing lipid peroxidation and DNA damage, and eventually death. ROS have pivotal roles in the effectiveness of mosquito resistance to insecticides. As pathogens have co-evolved with the arthropods that transmit them, the pathogens have devised ways to adapt to the physiological responses of the arthropods, even making use of their antioxidant response to evade ROS attacks of the host.

For the reasons mentioned above, it is important to consider the antioxidants or the antioxidant system as a potential target group in the development of drugs, vaccines, or other biological and chemical means to control the arthropods and their associated pathogens. In addition to the control aspects, this novel approach would address the vectorial capacity of these arthropods as well as their resistance to insecticides/acaricides. The use of phytochemicals has been a step in the right direction, but further understanding of the mechanism on how these phytochemicals work may be necessary to enable mass production globally.

## Figures and Tables

**Table 1:** Biological sources of ROS aside from blood meals.

Biological Activity	Reference
**Activity for reproduction and growth**	
Oogenesis	[36]
Embryonic development	[34,35]
Hatching and molting	[38]
Larval development	[34]
**Normal homeostasis**	
Flight activity	[37]
Nervous system activity	[16]
Cellular activity	[38]
**Pathogen infection**	
Microbial killing by ROS	[39]
Melanocytic encapsulation	[40]
Immune signaling	[41,42]
**Arthropod microbiome**	
Enterobacter production	[43]
**Arthropod control**	
Metabolism of acaricide/insecticide	[16]
Insecticide resistance	[44]
Phytochemical control	[45]
